# Deletion of *RAP1* affects iron homeostasis, azole resistance, and virulence in *Candida albicans*

**DOI:** 10.1128/msphere.00155-25

**Published:** 2025-04-23

**Authors:** Min-Chi Yang, Wei-Luen Huang, Hsuan-Yu Chen, Shin-Huey Lin, Yu-Shan Chang, Kuo-Yun Tseng, Hsiu-Jung Lo, I-Ching Wang, Chi-Jan Lin, Chung-Yu Lan

**Affiliations:** 1 Institute of Molecular and Cellular Biology, National Tsing Hua University 34881, Hsinchu, Taiwan; 2 Institute of Biotechnology, National Tsing Hua University, Hsinchu, Taiwan; 3 Institute of Molecular Biology, National Chung Hsing University, Taichung, Taiwan; 4 Taiwan Mycology Reference Center, National Institute of Infectious Diseases and Vaccinology, National Health Research Institutes, Miaoli County, Taiwan; 5 Department of Life Science, National Tsing Hua University 34881, Hsinchu, Taiwan; 6 School of Medicine, National Tsing Hua University 34881, Hsinchu, Taiwan; CNRS - Inserm - Université Côte d'Azur, Côte d'Azur, Nice, France

**Keywords:** *Candida albicans*, Rap1, iron acquisition, iron utilization, virulence, fluconazole susceptibility

## Abstract

Rap1 is a DNA-binding protein conserved from yeast to mammals for its role in telomeric maintenance. Here, to explore additional functions of *Candida albicans* Rap1, we performed RNA sequencing analysis. Experimental validations further showed that Rap1 plays a role in iron regulation, especially under low-iron conditions. Moreover, Rap1 was involved in iron acquisition and modulation of iron-related genes. Rap1 was found to be associated with fluconazole resistance in a low-iron condition. Finally, we demonstrated that the deletion of *RAP1* leads to reduced *C. albicans* virulence in a mouse model of infection. Together, this study reveals new functions of *C. albicans* Rap1, particularly in iron homeostasis, azole resistance, and virulence.

**IMPORTANCE:**

*Candida albicans* is an important pathogenic fungus that can cause superficial to life-threatening infections. Iron is essential for almost all organisms, yet it is highly restricted within the human host to defend against pathogens. To grow and survive in the iron-limited host environment, *C. albicans* has evolved multiple iron acquisition mechanisms. Understanding the regulation of iron homeostasis is, therefore, critical for elucidating *C. albicans* pathogenesis and virulence. This study explores the novel functions of *C. albicans* Rap1, with a focus on its contribution to iron acquisition and utilization. Our findings further highlight how iron availability impacts antifungal resistance and virulence through Rap1, providing insight into the complex iron regulatory machinery of *C. albicans*.

## INTRODUCTION

*Candida albicans* is a commensal fungus that normally inhabits the skin and mucosal surfaces of humans (1). However, *C. albicans* is also an opportunistic pathogen and can cause superficial to life-threatening invasive infections, especially in immunocompromised patients (2). Moreover, antifungal resistance of *C. albicans* holds another emergent threat to public health (3). Therefore, *C. albicans* has recently been included in the World Health Organization’s list of fungal priority pathogens, highlighting the urgent need for research and drug development for this important pathogen (4).

Iron is an essential micronutrient for *C. albicans* and its host, and it plays a crucial role in host-pathogen interactions (5). The human host employs various strategies to limit iron availability to *C. albicans*, known as nutritional immunity (6, 7). For example, host proteins such as transferrin and ferritin sequester iron, making it less available to *C. albicans*. To adapt to the iron-restricted host environment, *C. albicans* has developed three elaborate mechanisms for iron acquisition. First, *C. albicans* utilizes a reductive iron uptake system to acquire free iron, as well as sequestering iron from the host’s iron-binding proteins (8, 9). Ferric iron (Fe^3+^) is initially reduced to ferrous iron (Fe^2+^) by ferric reductases (5). The generated Fe^2+^ is oxidized again by multicopper ferro-oxidases, and Fe^3+^ is transported into the cell by the high-affinity iron transporter Ftr1 (5). Second, *C. albicans* also possesses a system that acquires iron from the host’s heme-binding proteins such as hemoglobin and serum albumin. This system is mediated by distinct hemophore proteins including Csa2, Rbt5, and Pga7 (10–12). Through a cascade of these proteins, heme is endocytosed into *C. albicans* cell and degraded by the vacuolar heme oxygenase Hmx1, releasing iron into the cytosol (10–12). Third, a siderophore-mediated iron acquisition has been identified in *C. albicans*. Although *C. albicans* does not produce its own siderophores, this pathogen uptakes xenosiderophores via a siderophore transporter, Sit1 (13).

Importantly, iron acquisition is also closely associated with pathogenicity and virulence of *C. albicans*. For example, the ferric reductase *CFL1*-deletion mutant shows attenuated virulence and reduced fungal burden in a mouse systemic infection model (14), and the mutant lacking *FTR1* is unable to establish systemic infection in mice (15). Moreover, Sit1 is required for epithelial invasion, while the *HMX1* null mutant exhibits decreased virulence and alters chemokine and cytokine expression in infected mice (13, 16). In addition, the reductive iron uptake system also affects biofilm formation, and iron deprivation induces hyphal development in *C. albicans* (17, 18). Finally, there is a link between iron and antifungal susceptibility (19). Iron depletion leads to increased susceptibility to antifungal agents in *C. albicans* due to alterations in membrane fluidity and permeability (20). The iron chelator deferasirox can reduce *C. albicans* invasion of oral epithelial cells and infection in murine oropharyngeal candidiasis (21).

Rap1 is a DNA-binding protein conserved in mammals, protozoa, and yeast (22). *Saccharomyces cerevisiae* Rap1 (ScRap1) regulates telomere homeostasis and length (23). Moreover, ScRap1 also possesses non-telomeric functions, including gene transcription and regulation of the ferric reductase *FRE1*, DNA repair, and ribosomal protein genes (24–26). In *C. albicans*, Rap1 is also crucial in controlling telomere length and structure (27, 28). Intriguingly, our recent study revealed that *C. albicans* Rap1 is also involved in cell wall integrity, biofilm formation, and virulence in a *Galleria mellonella* infection model (29). Nevertheless, our knowledge of other functions of *C. albicans* Rap1 remains limited.

In this study, we aimed to uncover previously unknown functions of Rap1 in *C. albicans*. Based on prior finding that ScRap1 is involved in regulating *FRE1* gene expression (25), we hypothesized that Rap1 might play roles related to iron availability. Our results showed that *RAP1* gene deletion affected cellular utilization of various iron sources and expression of iron acquisition-related genes. Additionally, we demonstrated that Rap1 contributes to fluconazole susceptibility, particularly under low-iron conditions. Notably, *RAP1* deletion also leads to attenuation of *C. albicans* virulence in a mouse model of systemic infection. Together, our findings reveal unexplored functions of Rap1 in *C. albicans*.

## RESULTS

### Iron-responsive transcriptome profiling of the *RAP1* deletion and wild-type strains

RNA-sequencing (RNA-seq) analysis was performed to identify differentially expressed genes (DEGs) in *C. albicans*. The *RAP1*-deleted (*rap1*Δ/Δ) and wild-type strains were pre-starved for iron by incubation with YPD medium containing 400 µM of the iron chelator bathophenanthrolinedisulfonate (BPS). Following this pre-treatment, cells were grown in YPD (high-iron condition) or YPD supplemented with 200 µM BPS (low-iron condition). These conditions were selected based on the differential activation of the iron transporters *FTR2* and *FTR1* under high- and low-iron environments, respectively, as previously described (15) and illustrated in Fig. S1.

**Fig 1:**
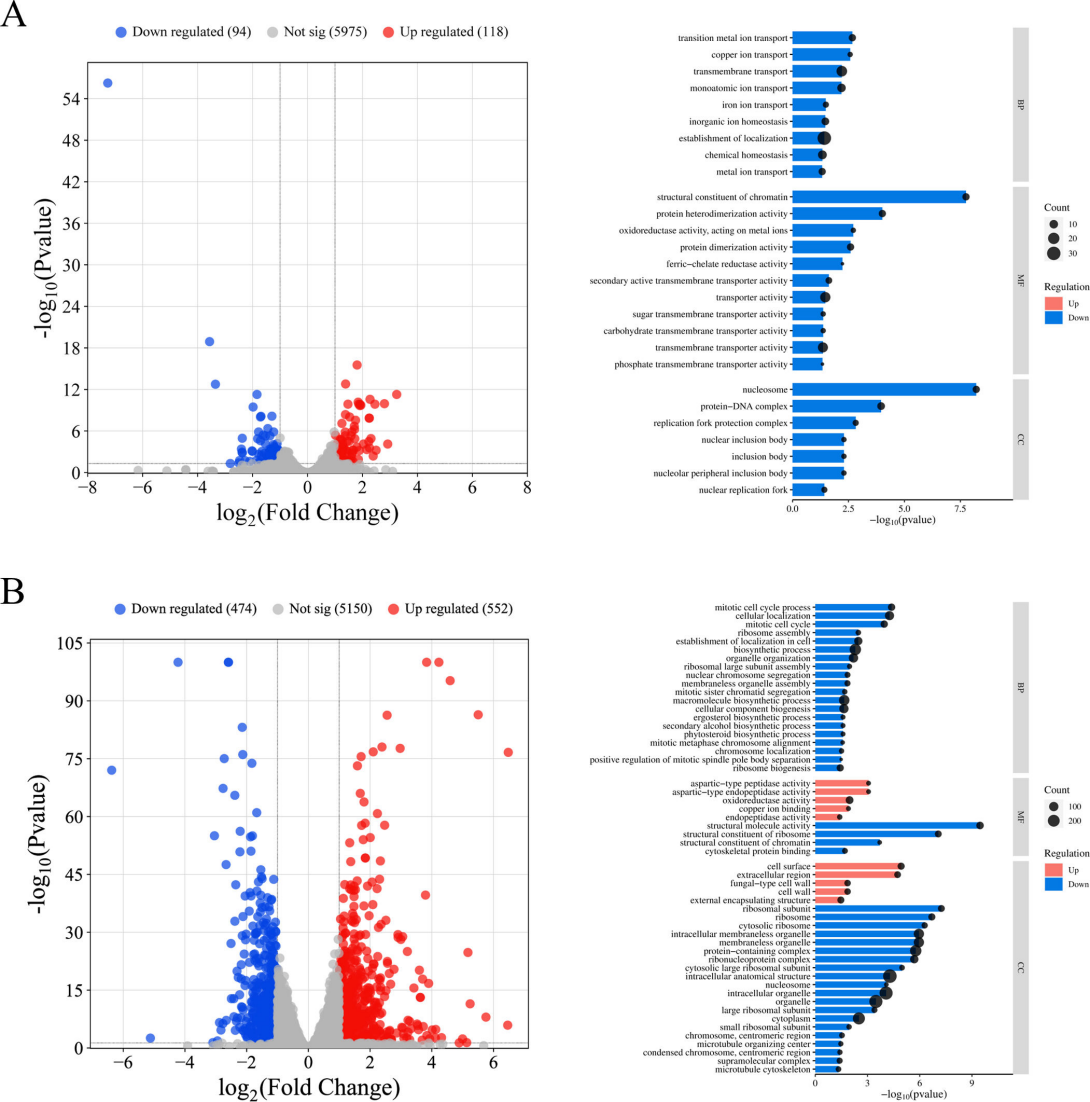
Transcriptome profiling of the *rap1*Δ/Δ mutant compared to the wild-type (WT) strain. (**A**) Volcano plot of RNA-seq data and bar graph of gene ontology (GO) enrichment analysis comparing gene expression in the *rap1*Δ/Δ mutant relative to the WT strain in YPD supplemented with 200 µM BPS (low iron) medium. GO terms significantly overrepresented among differentially expressed genes (DEGs) with a fold change ≥ 2 and adjusted *P*-value < 0.05. No GO terms were enriched among the upregulated genes. (**B**) Volcano plot and GO enrichment analysis of RNA-seq data for the *rap1*Δ/Δ mutant relative to WT in YPD (high iron) medium. Significantly upregulated and downregulated genes are shown in red and blue, respectively. “Not sig” indicates genes with no significant changes. BP, biological processes; CC, cellular components; MF, molecular functions.

DEGs were assessed using an adjusted *P*-value < 0.05 and an expression change of at least twofold (log2 fold change = 1). Under the low iron condition (YPD with 200 µM BPS), 118 genes were significantly upregulated and 94 genes were downregulated in the *rap1*Δ/Δ mutant. These changes are visualized in a volcano plot (Fig. 1A) and detailed in Table S1. Moreover, Gene Ontology (GO) analysis was performed. Although no GO terms were enriched among the upregulated genes, the downregulated genes were associated with multiple GO categories, including “iron ion transport,” “transition metal ion transport,” and “ferric-chelate reductase activity” (Fig. 1A; Table S2). For example, several ferric reductases and ferro-oxidase genes exhibited reduced expression in the *rap1*Δ/Δ mutant compared to the wild-type strain (Table S1). Interestingly, given the essential role of copper as a cofactor for ferro-oxidases (30, 31), a GO term associated with copper transport was also identified among the downregulated genes of the *rap1*Δ/Δ mutant (Tables S1 and S2).

Additionally, under the high iron condition, 552 genes were significantly upregulated, while 474 genes were downregulated in the *rap1*Δ/Δ mutant (Fig. 1B; Table S1). GO analysis classified these DEGs into multiple categories (Fig. 1B; Table S2), including “ergosterol biosynthetic process.” Overall, RNA-seq analysis suggests that *C. albicans* Rap1 plays pleiotropic roles in cellular homeostasis, including iron-responsive activities.

### Rap1 seems to mainly function in cells that are grown under low-iron conditions

**Fig 2:**
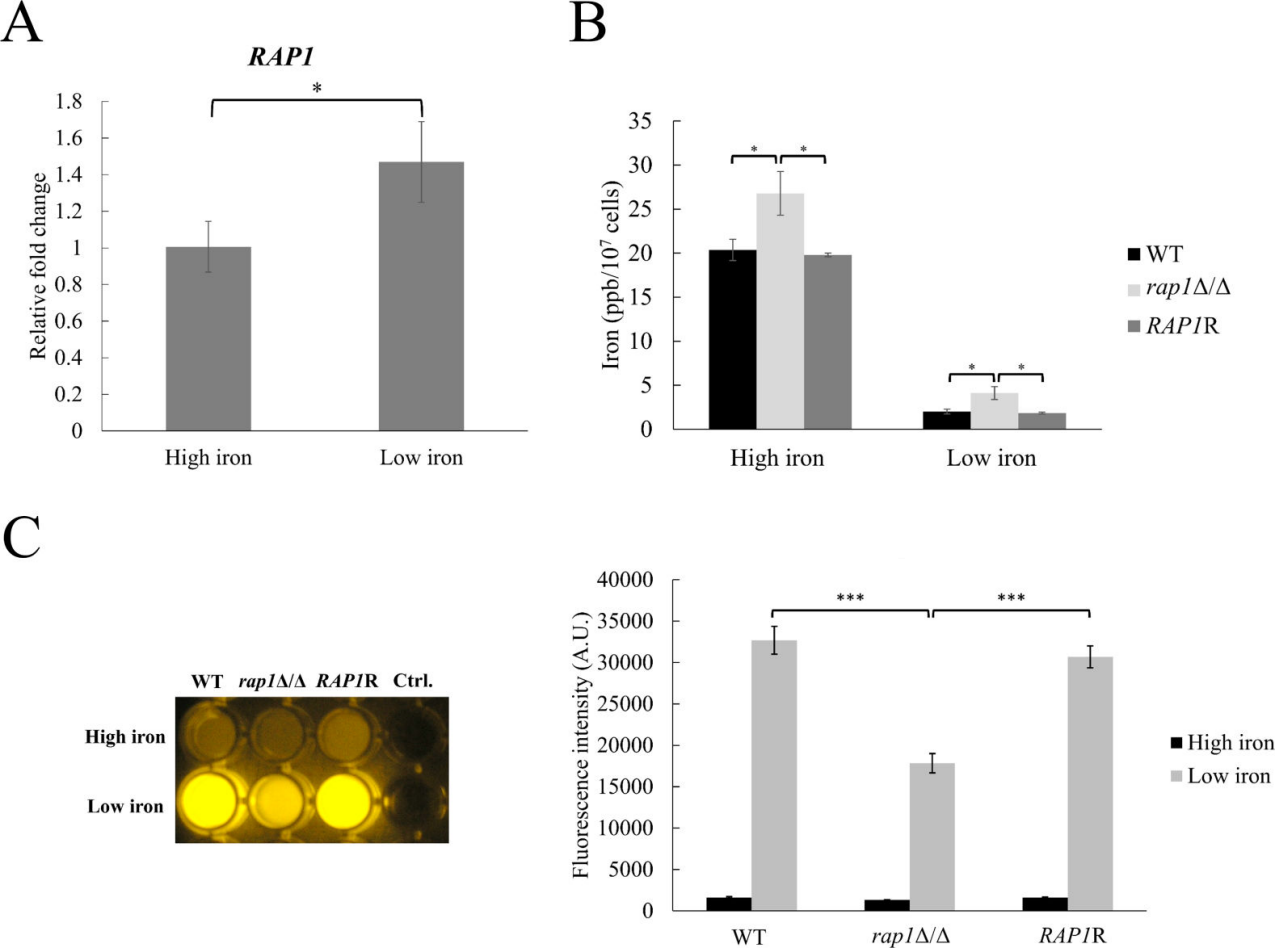
Rap1 functions in low iron conditions. (**A**) *RAP1* transcript level was determined by real-time qPCR. The *ACT1* transcripts were used as endogenous control. Data are collected from three independent experiments and presented as the mean ± standard deviation (SD). (**B**) Intracellular iron content was measured using ICP-MS. Data are collected from three independent experiments and presented as the mean ± SD. (**C**) Measurement of flavin production. Cells were cultured in high-iron (SC) or low-iron (NIM) media for 48 h. Left panel: culture supernatants were collected and photographed under UV excitation. The results from one of three replicates are displayed. Right panel: supernatants collected from cell culture were used to measure fluorescence upon excitation 450 nm and emission 530 nm. The values were normalized to cell numbers and presented as relative fluorescence intensity. WT, wild-type; *rap1*Δ/Δ, *RAP1*-deleted; *RAP1*R, *RAP1*-reintegrated strain. Data are collected from three independent experiments and presented as the mean ± SD. A.U., arbitrary units. *P < 0.05; ***P < 0.001.

To correlate Rap1 and iron-responsive functions, the effects of *RAP1* deletion on several iron-related traits were determined. In Fig. 2A, increased *RAP1* expression was detected in the low-iron condition using real-time quantitative PCR (qPCR). Moreover, although the details about intracellular iron trafficking in *C. albicans* are still not clear, cellular iron homeostasis is associated with the availability of environmental iron (14, 19). To further examine the relationship between Rap1 and iron response, the intracellular iron levels were measured using inductively coupled plasma mass spectrometry (ICP-MS). The results indicated that cells had much more intracellular iron contents when they grew in the high-iron YPD medium than in the low-iron YPD with BPS (Fig. 2B). In addition, the *rap1*Δ/Δ mutant had a higher intracellular iron level than that in the wild-type and *RAP1*-reintegrated control strains under either low- or high-iron condition (Fig. 2B). Thus, these data suggest that Rap1 likely functions in response to iron availability and acts preferentially toward the iron-limited condition. To test this hypothesis, flavin production was also assessed, as it is known that flavin production is induced by iron limitation (32). For the convenience of observation and spectrometric measurement, transparent yeast nitrogen base (YNB) non-iron medium (NIM) and synthetic complete (SC) medium were here used as the low- and high-iron condition, respectively (32). In Fig. 2C, the low secreted flavin levels of all tested strains were observed in the high-iron SC medium. However, secreted flavin levels were largely induced in the low-iron condition (NIM). Importantly, the *rap1*Δ/Δ mutant showed approximately 50% less flavin production compared to the control strains. Together, our findings indicate that deletion of *RAP1* affects iron-related traits, particularly in low-iron conditions.

### Rap1 is involved in iron utilization and iron acquisition-related gene expression

**Fig 3:**
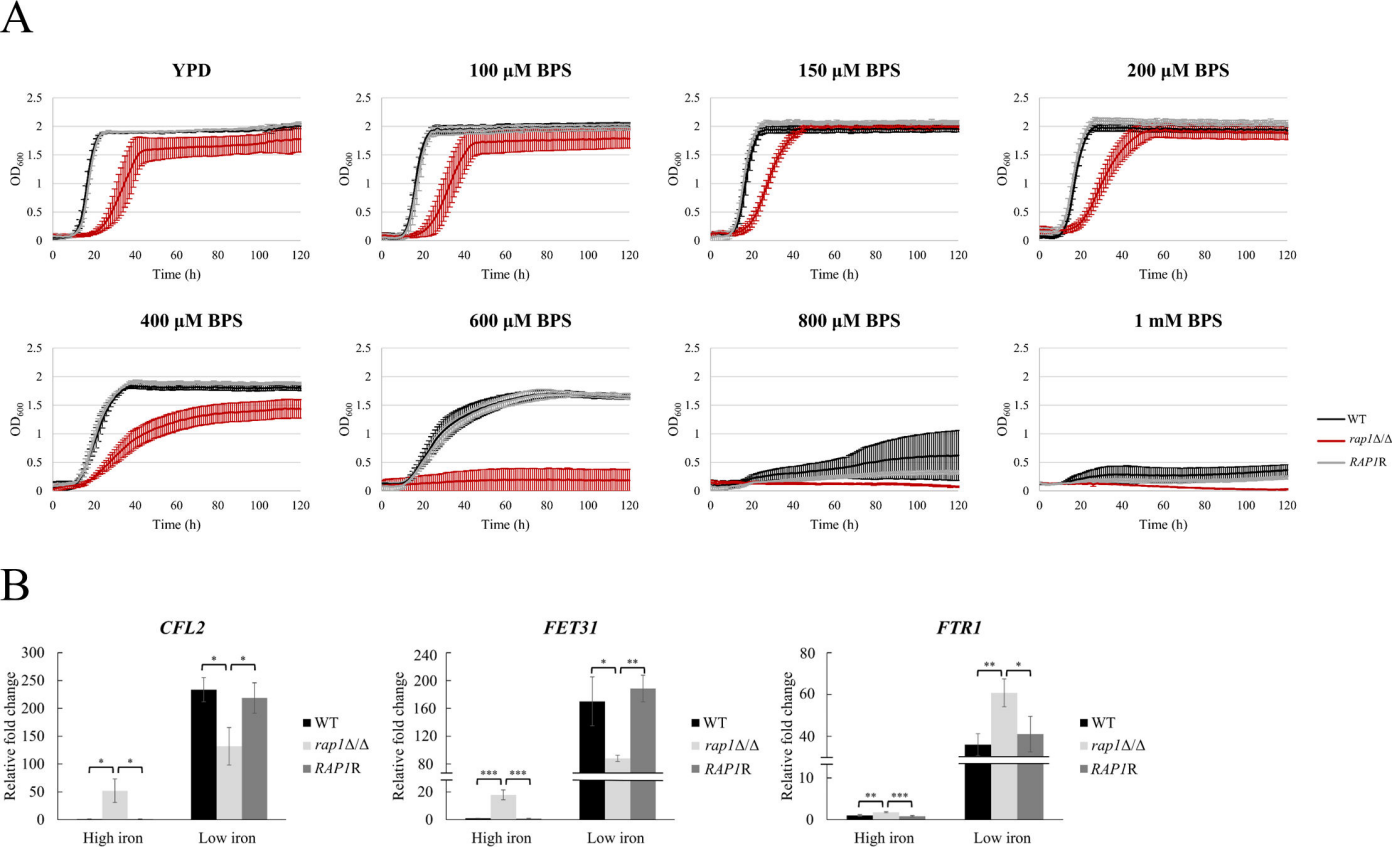
Deletion of *RAP1* affects utilization of different iron sources and expression of iron acquisition systems. (**A**) Iron pre-starved cells were inoculated into YPD broth supplemented with different concentrations of BPS. Data are collected from three independent experiments and presented as the mean ± SD. (**B**) Differential expression of genes in the reductive iron uptake system. Cells were harvested for RNA extraction, and gene expression was assessed by real-time qPCR. The *ACT1* transcripts were used as endogenous control. Data are collected from at least three independent experiments and presented as the mean ± SD. *P < 0.05; **P < 0.01; ***P < 0.001.

*C. albicans* has evolved to utilize diverse iron sources within the host environment via different iron acquisition systems (5). To further investigate iron-related functions of Rap1, iron utilization and expression of iron-acquisition genes were determined. For free iron utilization, iron-prestarved cells were grown in YPD broth with and without BPS (20, 32). As demonstrated in Fig. 3A, cell growth of all tested strains was inhibited under iron-limiting conditions, with increasing concentrations of BPS. However, the *rap1*Δ/Δ mutant was much more sensitive to iron limitation, particularly at 400 and 600 µM BPS, compared to control strains (Fig. 3A). These data suggest that the *rap1*Δ/Δ mutant is impaired in free iron acquisition, resulting in a more pronounced growth defect under low-iron conditions. To test this possibility, the expression of three representative genes of the reductive iron uptake system was assessed: the ferric reductase (*CFL2*), multicopper oxidase (*FET31*), and iron transporter (*FTR1*). In the low-iron condition, all three genes were largely induced (Fig. 3B). However, in the *rap1*Δ/Δ mutant, *CFL2* and *FET31* were downregulated, while *FTR1* expression was enhanced compared to control strains under the same condition (Fig. 3B).

### Rap1 is also associated with the acquisition and utilization of hemin and hemoglobin

Other than extracellular iron sources, *C. albicans* can also take up heme-bound iron of hemoglobin and hemin-containing iron from human erythrocytes. In this context, cell growth with hemoglobin and hemin was examined.

**Fig 4:**
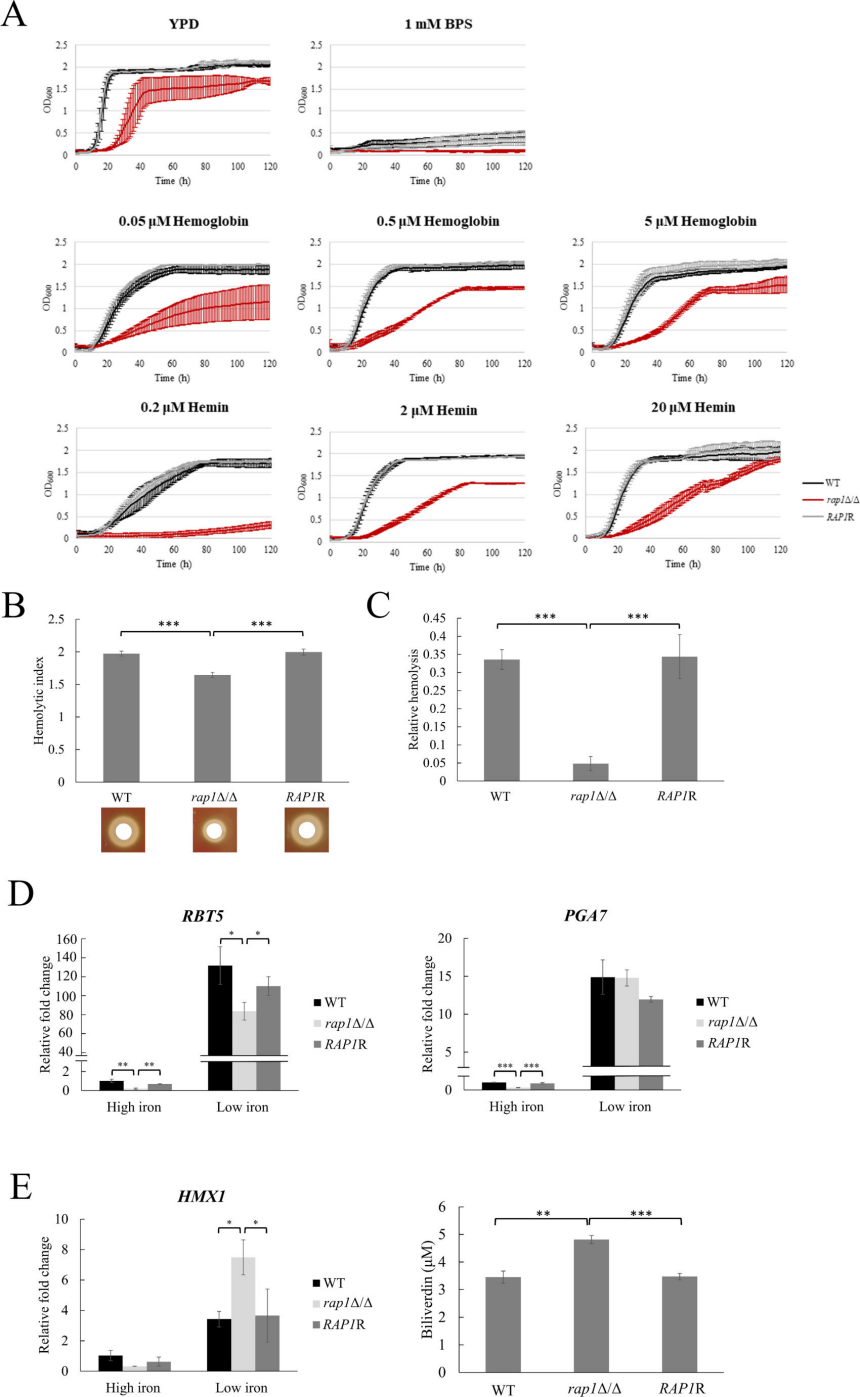
Rap1 is involved in heme uptake and utilization. (**A**) Iron pre-starved cells were inoculated into YPD broth supplemented with 1 mM BPS to prevent growth of *C. albicans* due to iron limitation. The indicated concentration of hemoglobin and hemin was added, and the cell growth curve was plotted. Data are collected from three independent experiments and presented as the mean SD. (**B**) Blood agar assay. Cells were spotted onto blood agar and incubated at 37°C, 5% CO_2_ for 24 h. Zones of beta hemolysis were assessed (lower panel). The hemolytic index was calculated from five independent experiments and presented as the mean ± SD (upper panel). (**C**) Hemolysis liquid assay. Yeast cells were cultured with sheep red blood cells at 37°C, 5% CO_2_ for 24 h, and the hemolytic activity was measured by absorbance at 414 nm. Results were collected from five independent experiments and presented as the mean ± SD. (**D**) Expression of the heme transporter genes. Cells were harvested for RNA extraction, and gene expression was assessed by real-time qPCR. The *ACT1* transcripts were used as endogenous control. Data are collected from at least three independent experiments and presented as the mean ± SD. (**E**) Expression and activity of the heme oxygenase Hmx1. Left panel: expression of the heme oxygenase gene was measured using real-time qPCR. The *ACT1* transcripts were used as endogenous control. Data are collected from at least three independent experiments and presented as the mean ± SD. Right panel: cells were cultured in the medium containing hemoglobin for 12 h and collected. The heme oxygenase activity was determined by measuring absorption of biliverdin at 666 nm. Data are collected from three independent experiments and presented as the mean ± SD. *P < 0.05; **P < 0.01; ***P < 0.001.

In Fig. 4A, all tested strains exhibited severe growth defects in YPD broth containing 1 mM BPS. These growth defects of the wild-type and *RAP1*-reintegrated control strains were easily recovered by adding different concentrations of hemoglobin and hemin. Although hemoglobin and hemin could also rescue the growth of the *rap1*Δ/Δ mutant; however, the mutant exhibited a growth delay in the presence of these iron sources compared to its growth in YPD broth alone (Fig. 4A).

To access heme iron from the host, *C. albicans* secretes hemolytic factors to lyse host red blood cells (RBCs) (33). Accordingly, the hemolytic activity of the *rap1*Δ/Δ mutant was further examined using blood agar and liquid hemolysis assay. In the agar assay, cells were spotted on agar plates containing 7% sheep blood, and hemolytic zones were recorded (34). As demonstrated in Fig. 4B (lower panel), all test strains formed β-hemolysis (translucent halo) and α-hemolysis (dark green halo) that represent complete and incomplete RBC degradation, respectively. Interestingly, *RAP1* deletion resulted in reduced hemolytic zones compared to the control strains. The hemolytic index was further measured as previously described (34). Evidently, the *rap1*Δ/Δ mutants had a lower hemolytic index than the control strains (Fig. 4B, upper panel). Furthermore, the liquid hemolysis assay was performed by co-culturing *C. albicans* and sheep RBCs, and concentrations of extracellular hemoglobin levels were measured (33). The results showed that the *rap1*Δ/Δ mutant possesses a lower hemolytic activity (Fig. 4C).

Once hemoglobin is released from RBCs, *C. albicans* uptakes heme via heme transporters on the cell envelope. Notably, among these heme transporters, *RBT5* is one of the most highly induced genes in *G. mellonella* (35), and Rbt5-specific antibodies are notable in candidemia patients (36). Moreover, the deletion of *PGA7* attenuates virulence in a murine model of disseminated candidiasis (11). Thus, *RBT5* and *PGA7* expression was measured, and the results found that these genes were highly induced in the low-iron condition (Fig. 4D). Moreover, in the low-iron condition, *RBT5* was downregulated in the *rap1*Δ/Δ mutant compared to the control strains (Fig. 4D).

After transferring across the cell envelope, heme is degraded by the heme oxygenase Hmx1 to release iron ions (37, 38). As expected, *HMX1* expression was induced in the low-iron condition (Fig. 4E). Moreover, the *rap1*Δ/Δ mutant exhibited a higher *HMX1* gene expression than the control strains in the low-iron condition (Fig. 4E). Finally, the activity of heme oxygenase was also assessed by growing cells in medium containing hemoglobin. As shown in Fig. 4E, the Hmx1 activity in the *rap1*Δ/Δ mutant was elevated compared to the controls, by measuring concentrations of biliverdin, a waste product of heme degradation (16).

In sum, our results showed that *RAP1* deletion leads to alterations in iron utilization and iron acquisition gene expression, suggesting *C. albicans* Rap1 plays a role in iron homeostasis.

### *RAP1* deletion also affects fluconazole susceptibility in response to iron conditions

Azoles are commonly used antifungal drugs by targeting the sterol 14α-demethylase Erg11 to inhibit ergosterol biosynthesis, thereby blocking fungal cell membrane formation (3, 39). Disrupting ergosterol biosynthesis can also cause accumulation of toxic sterols catalyzed by the sterol C5-desaturase Erg3 (3, 39). Furthermore, iron availability affects fluconazole susceptibility in *C. albicans* (20). Interestingly, our RNA-seq data revealed differential expression of ergosterol biosynthesis genes in the *rap1*Δ/Δ mutant compared to the wild-type strain grown in YPD (Table S1). This observation raises the question of whether a relationship exists between Rap1 and fluconazole susceptibility with respect to iron availability.

**Fig 5:**
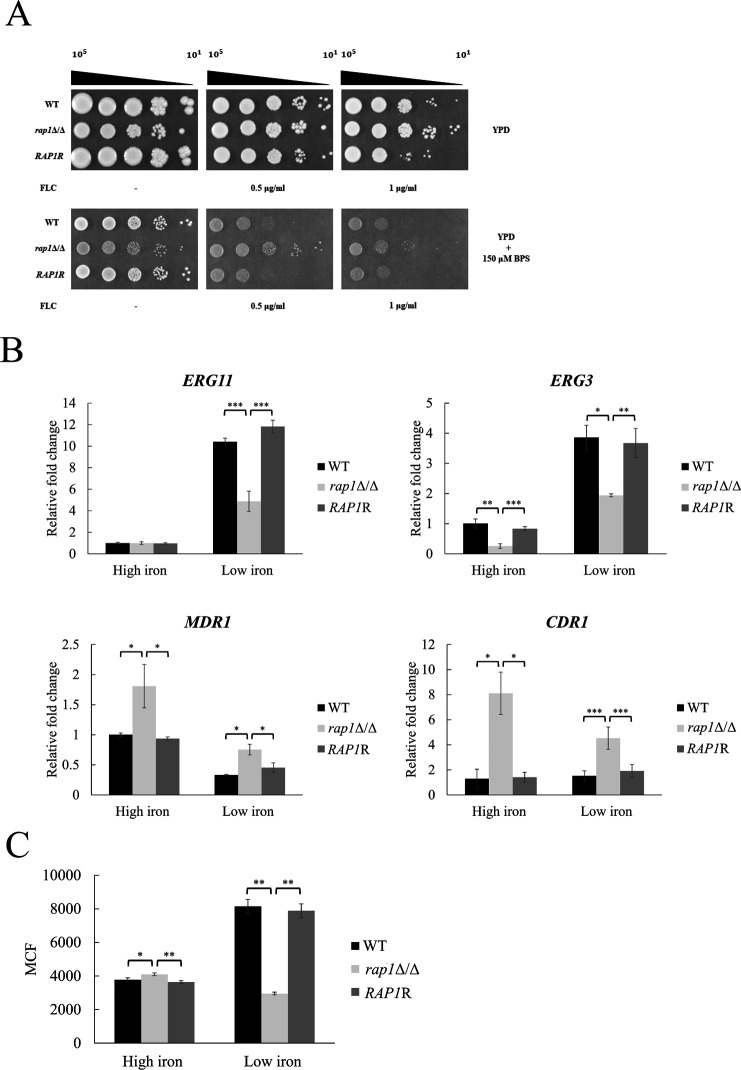
The *RAP1* deletion mutant is more resistant to fluconazole in response to iron condition. (**A**) Fluconazole spot assay. Iron pre-starved cells were spotted on YPD agar plates in the presence or absence of BPS and fluconazole (FLC). (**B**) Expression of ergosterol biosynthesis and drug pump genes. Exponential phase cells were harvested for RNA extraction, and gene expression was assessed by real-time qPCR. The *ACT1* transcripts were used as endogenous control. Data are collected from at least three independent experiments and presented as the mean ± SD. (**C**) The efflux activity of drug pump was measured using the dye Nile red. MCF, mean channel fluorescence. Data are collected from three independent experiments and presented as the mean ± SD. *P < 0.05; **P < 0.01; ***P < 0.001.

To test this possibility, fluconazole susceptibility was determined using agar and broth microdilution methods. In the spot assay, cell growth of all tested strains showed no significant difference in YPD agar plates. However, the *rap1*Δ/Δ mutant displayed a slight resistance to fluconazole (1 µg/mL) than the control strains (Fig. 5A). Apparently, the *rap1*Δ/Δ mutant was even much more resistant to fluconazole than the control strains in YPD agar with BPS added (Fig. 5A). Similarly, the broth microdilution assay demonstrated that all tested strains had higher minimum inhibitory concentration 50 (MIC50) in YPD compared to that in YPD containing BPS (Table 1). Moreover, the *rap1*Δ/Δ mutant had higher MIC50, suggesting more resistance of the mutant to fluconazole than the control strains (Table 1).

**TABLE 1:** Fluconazole susceptibility of *C*. *albicans*^*a*^

Condition	Strains	24 h MIC50 (μg/mL)	48 h MIC50 (μg/mL)
YPD	WT	0.5	0.5
	*rap1*Δ/Δ	1	1
	*RAP1*R	0.5	0.5
YPD + 150 µM BPS	WT	0.25	0.25
	*rap1*Δ/Δ	1	0.5
	*RAP1*R	0.25	0.25

^*a*^ MIC50, minimum inhibitory concentration 50. WT, wild-type (SC5314) strain; *rap1*Δ/Δ, *RAP1*-deleted strain; *RAP1*R, *RAP1*-reintegrated strain.

Several mechanisms are known to contribute to fluconazole resistance. For example, overexpression or point mutations of the *ERG11* gene, and mutations in Erg3 by preventing toxic sterol accumulation can all lead to drug resistance (40–43). To further investigate if fluconazole resistance in the *rap1*Δ/Δ mutant is linked to ergosterol biosynthesis in response to iron availability, the expression of *ERG11* and *ERG3* was measured from cells grown in the low- and high-iron condition. Expression of *ERG11* and *ERG3* genes was largely induced in the low-iron condition (Fig. 5B). Moreover, the *rap1*Δ/Δ mutant showed a lower expression level of *ERG11* and *ERG3* than the control strains, particularly under the low-iron condition (Fig. 5B).

Another mechanism of azole resistance involves overexpression of efflux pump genes. Therefore, real-time qPCR analysis was performed, and the results indicated that the expression of *CDR1* and *MDR1* was elevated in the *rap1*Δ/Δ mutant under both low- and high-iron conditions (Fig. 5B). Finally, since Nile red is a known fluorescent substrate for Cdr1 and Mdr1 transporters, efflux pump activity was further assessed by measuring Nile red accumulation in the cells (44). In Fig. 5C, Nile red accumulation was decreased particularly in the *rap1*Δ/Δ mutant compared to the control strains under low-iron condition, suggesting a stronger efflux activity in the *rap1*Δ/Δ. Together, the deletion of *RAP1* led to fluconazole resistance under low-iron condition, and this phenotype is associated with alterations in ergosterol biosynthesis and drug efflux pumps.

### Loss of *RAP1* impedes *C*. *albicans* virulence in a mouse model of systemic infection

**Fig 6:**
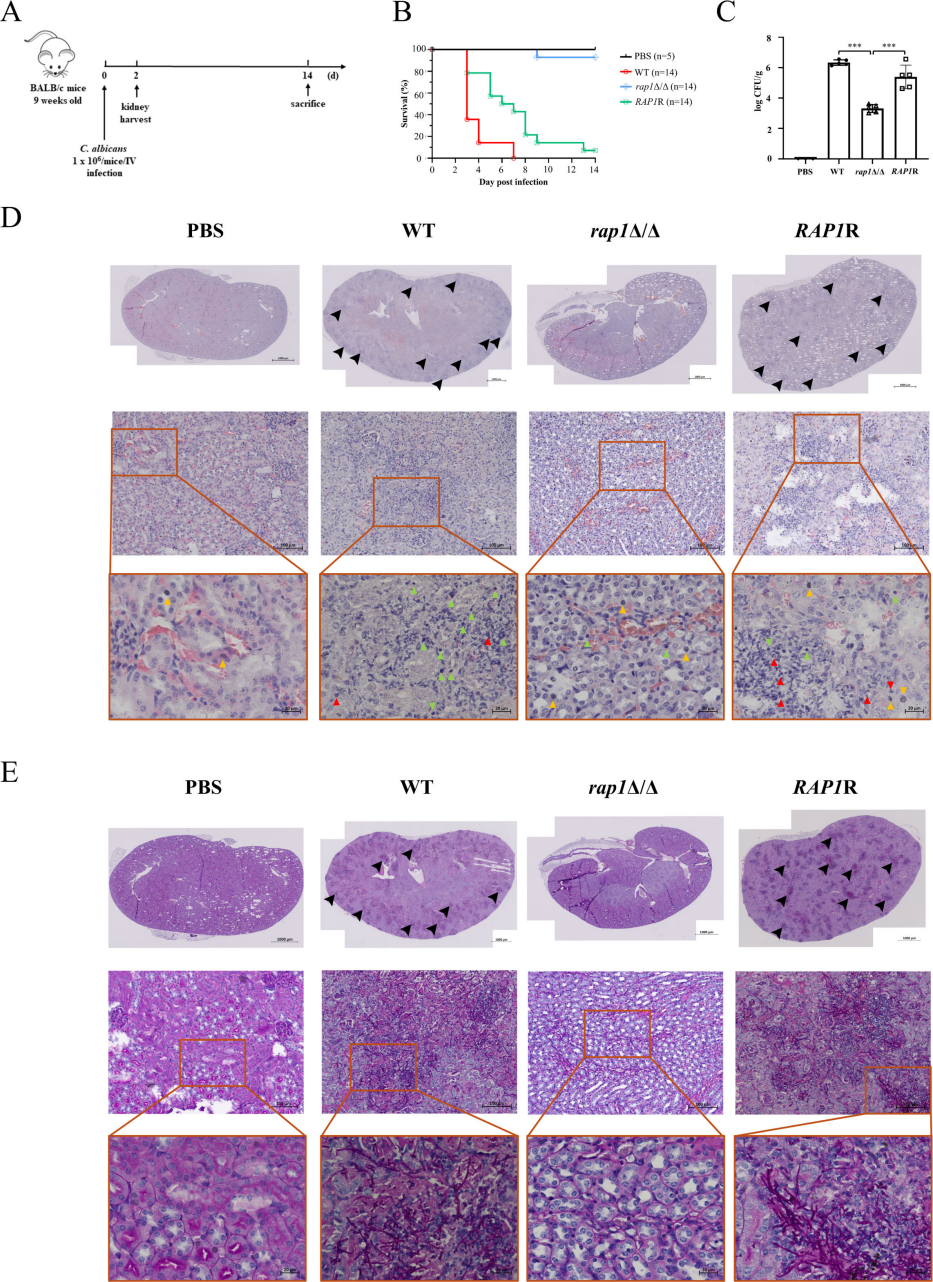
Deletion of *RAP1* attenuates *C. albicans* virulence. (**A**) Diagram of the experimental design. Nine-week-old BALB/c mice (*n* = 19) were injected via the tail vein with 1 × 10^6^ *C. albicans* cells, including wild-type (WT), *rap1*Δ/Δ mutant, and *RAP1*-reintegrated strains (*RAP1R*). The mice kidneys were collected on the second day post-infection for fungal burden and histological examination. The rest of the mice were euthanized at 14 days post-infection. (**B**) Assessment of the survival rate of mice. The number of surviving mice was plotted against time. (**C**) Assessment of fungal burden. Mice were sacrificed at 2 days post-infection, and kidneys were collected and homogenized. Homogenates were used for counting the number of colony-forming unit (CFU) by plating on YPD agar plates. Results are expressed as log CFU/g kidney and expressed as mean ± SD from mice infected with each *C. albicans* strain. The H&E (**D**) and PAS (**E**) staining. Upper panel: images of stained kidney samples were captured at 100× magnification. Arrowheads indicate inflammatory foci and highlight leukocyte infiltration in the H&E staining and fungal colonization in the PAS staining. Middle and lower panel: images of stained kidney samples were captured at 400× and 630× magnification. In the lower panel of H&E staining, basophil, lymphocyte, and neutrophil are represented by yellow, green, and red arrowheads, respectively. Scale bars denote magnification. The representative images from five infected mice with similar results are shown.

The human body maintains extremely low levels of free iron to defend against pathogens, and iron acquisition is closely associated with the virulence of *C. albicans* (45). Given that Rap1 is relevant to iron homeostasis, we, therefore, used a mouse model of systemic candidiasis and histological analysis to determine the role of Rap1 on *C. albicans* virulence and pathology (Fig. 6A). The wild-type and *RAP1*-reintegrated strains had high virulence, by exhibiting rapid death of the mice beginning on the second day post-infection, and almost all mice in these two groups died during the experiment (Fig. 6B). Conversely, more than 90% of mice injected with the *rap1*Δ/Δ mutant remained alive within 14 days post-infection (Fig. 6B). The death rate of mice infected with the *rap1*Δ/Δ mutant was significantly lower than that of the wild-type (*P*-value < 0.0001) and *RAP1*-reintegrated strain (*P*-value = 0.0014), indicating that the deletion of *RAP1* attenuated *C. albicans* virulence.

To determine organ colonization, kidneys were collected. The kidney fungal burden was significantly lower in mice infected with the *rap1*Δ/Δ mutant than that with the infection of the wild-type and *RAP1*-reintegrated control strains (Fig. 6C). Histological examinations of kidneys were also performed by hematoxylin and eosin (H&E) staining. The examination demonstrated that kidneys from mice infected with the control strains displayed inflammation with leukocytes, whereas no obvious cell infiltration was observed in that injected with the *rap1*Δ/Δ mutant and PBS (Fig. 6D). Finally, Periodic acid-Schiff (PAS) staining was also applied to detect fungal invasion (Fig. 6E). In the kidney sections examined, the control cells were observed as filamentous forms surrounded by leukocytes, while little to none of the *rap1*Δ/Δ mutant was observed. Collectively, these findings indicate the involvement of Rap1 in *C. albicans* virulence and pathogenicity.

## DISCUSSION

Iron is essential but potentially toxic to cause oxidative stress (46). Importantly, the host uses nutritional immunity to limit iron availability to pathogens (6, 7). Thus, maintaining iron balance by regulating iron homeostasis is crucial for both host and pathogen.

Rap1 is a protein that participates in telomere maintenance (22, 28, 47). Notably, *C. albicans* Rap1 lacks the C-terminal domain implicated in telomere regulation of ScRap1 (28). Moreover, *ScRAP1* is an essential gene, whereas *RAP1* is not essential for cell viability of *C. albicans* (28). In addition, our recent studies demonstrated that *C. albicans* Rap1 regulates cell wall integrity, biofilm formation, and oxidative stress response (29, 48). The *rap1*Δ/Δ mutant also modulated macrophage-pathogen interaction and showed attenuated virulence in a *G. mellonella* infection model (29). These results highlighted the significant role of Rap1 in *C. albicans* pathogenicity and virulence. In this work, we explored other functions of *C. albicans* Rap1 and found its connection with cellular response to iron.

Several iron-related traits were disclosed to correlate functions of Rap1 with iron response. Although the action of Rap1 under high-iron conditions could not be excluded, *RAP1* deletion affected those traits more obviously under conditions of low iron (Fig. 2A through C). A specific example is the strong effect of *RAP1* deletion on low-iron-induced flavinogenesis (Fig. 2C). Flavins such as flavin-adenine dinucleotide (FAD), flavin mononucleotide (FMN), and riboflavin (RF) are responsible for electron transfer and redox processes (49, 50). FAD and FMN are essential for forming flavoproteins, which are involved in the respiratory chain and fatty acid oxidation (51, 52). Recently, RF production and its regulation are also considered a novel antifungal drug target (53). Interestingly, RF is involved in ferric iron reduction, thereby promoting iron acquisition (54, 55). Once reduced, flavin-bound reductases release iron from ferric-siderophores, driving iron assimilation (56, 57). Although the detailed mechanisms of inducible flavinogenesis remain unclear, *C. albicans* flavinogenesis is controlled by different transcription factors in response to iron availability (32, 53, 58–60). For example, Tup1 is a global regulator controlling different features including filamentation and biofilm formation (61–64), whereas Hap43 and Sef1 are required for low-iron response. Interestingly, flavin production was largely decreased and completely abrogated in iron depletion in the *TUP1*-deleted and *HAP43*-deleted mutant, respectively (32). In addition, overexpression of *SEF1* resulted in higher RF secretion under an iron-replete condition (59), while deletion of *SEF1* reduced flavin production under iron deprivation (53). Finally, overexpression of Irf1, a regulator for morphogenesis and iron homeostasis, also led to higher flavin production (60). To determine whether a relationship exists between Rap1 and other regulators in iron-responsive flavinogenesis would be an interesting direction for future research.

The present study also linked Rap1 to the utilization of free iron and its impact on the reductive iron uptake gene expression (Fig. 3A and B). A regulatory circuit consisting of three transcription factors Sef1, Hap43, and Sfu1 has been previously identified to control iron homeostasis in *C. albicans* (65). Sef1 and Hap43 function under low-iron conditions, whereas Sfu1 is a high-iron regulator (32, 66–69). Sef1 promotes iron acquisition and activates *HAP43* expression (66, 67). Reversely, Hap43 represses iron utilization genes and regulates intracellular iron homeostasis (32, 69). Additionally, Hap43 represses Sfu1, which prevents toxicity of iron overload by repressing Sef1 and iron uptake genes (68–70). Therefore, determining whether Rap1 mutually interacts with Sef1, Hap43, and Sfu1 in iron regulation is also intriguing. Furthermore, Rap1 seems to be involved in hemin and hemoglobin utilization. The *rap1*Δ/Δ mutant exhibited reduced hemin and hemoglobin utilization, as well as reduced hemolysis (Fig. 4A through E). *C. albicans* secretes hemolytic factors to lyse host RBCs and release iron from hemoglobin (71, 72). Recently, candidalysin is identified as a hemolytic factor of *C. albicans* (33). Candidalysin is a peptide derived from a precursor protein Ece1 and can form membrane pores to damage host cells (73–75). Interestingly, our RNA-seq data showed an upregulation for the *ECE1* expression in the *rap1*Δ/Δ mutant compared to the wild type in YPD broth (Table S1). Therefore, whether Rap1 can affect candidalysin production in response to iron conditions is of interest for further investigation.

Fluconazole represents one of the first-line anti-*Candida* drugs (76, 77). Azoles block ergosterol biosynthesis, and complex links between iron and azole susceptibility in *Candida* species have been reported (19). For example, iron acts as a cofactor for several ergosterol biosynthesis-related enzymes including Erg11 and Erg3, and ergosterol levels are tightly regulated by iron availability (78). Moreover, iron depletion leads to reduced ergosterol production, resulting in higher membrane fluidity and enhanced azole susceptibility (20). Finally, the deletion of the iron permease gene *FTR1* makes cells more susceptible to fluconazole (20), and heme transporter gene *RBT5* is upregulated in cells treated with fluconazole (79). In this study, the *rap1*Δ/Δ mutant was more resistant to fluconazole and had lower expression of *ERG11* and *ERG3*, particularly under low-iron condition (Fig. 5A and B). Thus, alterations in membrane lipid phase and asymmetry membrane may occur in this mutant, impacting membrane fluidity and passive diffusion to contribute to azole resistance (80, 81). In addition, the *rap1*Δ/Δ mutant appears to have higher expression of drug pump genes in both low- and high-iron condition (Fig. 5B). Of note, the *rap1*Δ/Δ mutant also showed a higher efflux pump activity than the control strains in the low-iron condition (Fig. 6D). Therefore, our findings suggest an intricate association between Rap1, iron, and fluconazole.

Finally, although Rap1 impacts iron homeostasis and virulence in this study, it has also been connected to multiple other functions in *C. albicans* (27–29, 48). In humans, a correlation exists between iron homeostasis, telomere maintenance, and diseases, involving complex processes such as oxidative stress and DNA damage (82, 83). Interestingly, the role of telomeric and sub-telomeric structures in fungal pathogens has become a focus of recent studies (84). Since Rap1 exerts pleiotropic functions and is involved in telomere maintenance in *C. albicans*, it is possible that some or all of its observed effects are indirect, potentially involving telomere-associated factors such as genome structure and stability. However, further studies are needed to clarify these mechanisms.

## MATERIALS AND METHODS

### Strains and growth conditions

*C. albicans* strains used in this study are listed in Table S3. Cells were routinely maintained at −80°C and plated on YPD plates (1% yeast extract, 2% peptone, 2% glucose, and 1.5% agar). A single colony was inoculated into respective medium and incubated overnight (~16 h) at 30°C with shaking (180 rpm). All reagents were purchased from Sigma-Aldrich (St. Louis, MO, USA) unless indicated otherwise.

### RNA-seq analysis

Methods for RNA-seq and its data analysis are included in the supplemental material.

### Iron-dependent growth analysis

To assess iron-dependent cell growth, a single colony was inoculated into YPD and grown overnight at 30°C. The overnight cultures were subcultured in YPD containing 400 µM BPS and grown at 30℃ with shaking (180 rpm) for 24 h to deplete intracellular iron content. These iron pre-starved cultures were harvested, washed with sterile double-distilled water (ddH_2_O), and subcultured into high- and low-iron media, respectively. Cells were inoculated in a 96-well flat microplate and incubated at 30℃ for 5 days. Cell growth was measured using a SPECTROstar Nano (BMG Labtech, Ortenberg, Germany).

### RNA extraction and reverse transcription real-time qPCR

Iron pre-starved cells were cultured in high- and low-iron media for 5 h at 30℃. Total RNA extraction, RT for cDNA synthesis, and real-time qPCR were performed as previously described (32). The primers used are listed in Table S4. All experiments were performed in duplicate, with at least three biological replicates for each strain, and the relative fold change in gene expression was calculated using the 2^−ΔΔ^CT method (85).

### Measurement of intracellular iron level

Intracellular iron level was measured as previously described (86). Iron-prestarved cells were cultured in high- and low-iron media for 5 h at 30℃. To quantify intracellular iron level, cells were resuspended in 200 µL of 70% nitric acid and incubated at room temperature for 24 h, and 800 µL of ddH_2_O was added. The resulting mixture was analyzed using a Thermo Fisher Scientific iCAP TQ ICP-MS. The iron contents were normalized to cell numbers for each sample.

### Measurement of flavin production

To measure iron starvation-induced flavin production, NIM was used as previously described (32, 68). The NIM contains 0.17% YNB without Fe and Cu, 0.079% complete supplement mixture, 2% glucose, 0.5% ammonium sulfate, and 0.25 µM CuSO_4_ and is supplemented with 100 µM BPS. Briefly, cells were iron-starved in NIM at 30°C with shaking for 2 days, and cell densities were determined. Then, supernatants were collected, and the fluorescence was measured (emission: 530 nm; excitation: 450  nm). The relative ratio of fluorescence was normalized to the cell densities for each strain. Moreover, the supernatants of each sample were photographed under UV light.

### Measurement of hemolytic activity

Blood agar plates were prepared as previously described (36) with some modifications. Briefly, a single colony was inoculated in Sabouraud dextrose broth (1% peptone and 4% glucose, pH 5.6) and incubated at 37℃ overnight. Cells were collected and resuspended in sterile ddH_2_O to a concentration at OD_600_ of ~10. Ten microliters of each diluent was spotted onto sugar-enriched blood agar plates, which were prepared by adding 7 mL of fresh sheep blood into 100 mL Sabouraud dextrose containing 3% (wt/vol) glucose and agar (final pH 5.6 ± 0.2). After incubation at 37℃ in 5% CO_2_ for 24 h, the hemolytic index was calculated using the following formula: hemolytic index = β-hemolysis diameter/colony diameter.

The liquid hemolysis assay was performed as previously described (35) with some modifications. Sheep RBCs and *C. albicans* cells were mixed in a 1:1 ratio in RPMI 1640 medium (Gibco) and incubated with horizontal shaking (50 rpm) at 37℃ in 5% CO_2_ for 24 h. After centrifugation, the supernatant was transferred to a 96-well microplate to measure absorbance (Abs) at 414 nm. Relative hemolysis was defined as follows:$\;relative\;hemolysis=\;\frac{Abs\;414\;nm\;\left(sample-vehicle\;control\right)}{Abs\;414\;nm\;\left(full\;lysis\;control\;-vehicle\;control\right)}$.

### Measurement of heme oxygenase activity

Activity of heme oxygenase was determined by measuring biliverdin production as previously described (16). Iron pre-starved cells were harvested, washed, and resuspended in ddH_2_O. Cell suspension was inoculated into YPD broth containing 25 µM hemoglobin and 1 mM BPS. After incubation for 12 h, 6 × 10^9^ cells were collected, resuspended in 1 mL methanol, vortexed for 30 s, and centrifuged (5,000 × *g*) at room temperature. Biliverdin concentrations were determined by absorbance measurement at 666 nm and calculated using a coefficient of 14,400 M^−1^cm^−1^ (87)

### Virulence assay

Methods for virulence assays are included in the supplemental material.

### Fluconazole susceptibility testing

Fluconazole susceptibility was determined by agar and broth microdilution assay. Iron-prestarved cells were 10-fold diluted with sterile ddH_2_O and spotted onto YPD agar plates with and without BPS and fluconazole. The plates were incubated at 30℃ for 3 days. Broth microdilution assay was determined according to the Clinical and Laboratory Standards Institute’s M27-A3 guideline (88) with some modifications. Iron-prestarved cells were inoculated into 96-well flat-bottomed plates in YPD medium with or without BPS and different concentrations of fluconazole. After incubation at 30℃ for 24 and 48 h, MIC50 was defined as the drug concentrations capable of reducing cell turbidity by more than 50%.

### Measurement of Nile red accumulation

The accumulation of Nile red was determined as previously described (44). Iron-prestarved cells were cultured in high- and low-iron media for 5 h at 30℃. Cells were collected, washed, resuspended in PBS containing 7 µM of Nile red, and incubated at 30°C for 30 min. One hundred microliters of cell suspension was transferred to a black 96-well microplate, and the fluorescence intensity of Nile red was detected using a CLARIOstar Plus microplate reader (BMG Labtech, Ortenberg, Germany).

### Statistical analysis

The two-tailed Student’s *t*-test was used to determine significant differences between samples. Statistical significance was indicated with a *P*-value < 0.05.

## Data Availability

The data presented in this study are available within the article and the supplemental material. RNA-seq data were submitted to the NCBI Sequence Read Archive (accession numbers PRJNA1148770 and PRJNA1187199).

## SUPPLEMENTAL MATERIAL

Supplemental material msphere.00155-25-s0001.docx Fig. S1, Table S1 and S2 captions, Tables S3 and S4, and supplemental text.

Table S1 msphere.00155-25-s0002.xlsx List of DEG.

Table S2 msphere.00155-25-s0003.xlsx GO categories.

ASM does not own the copyrights to Supplemental Material that may be linked to, or accessed through, an article. The authors have granted ASM a non-exclusive, world-wide license to publish the Supplemental Material files. Please contact the corresponding author directly for reuse.

## References

[1] Limon JJ, Skalski JH, Underhill DM. 2017. Commensal fungi in health and disease. Cell Host Microbe 22:156–165. doi:10.1016/j.chom.2017.07.002 28799901 PMC5573128

[2] Lopes JP, Lionakis MS. 2022. Pathogenesis and virulence of Candida albicans. Virulence 13:89–121. doi:10.1080/21505594.2021.2019950 34964702 PMC9728475

[3] Lockhart SR, Chowdhary A, Gold JAW. 2023. The rapid emergence of antifungal-resistant human-pathogenic fungi. Nat Rev Microbiol 21:818–832. doi:10.1038/s41579-023-00960-9 37648790 PMC10859884

[4] WHO. 2022. WHO fungal priority pathogens list to guide research, development and public health action. World Health Organization

[5] Fourie R, Kuloyo OO, Mochochoko BM, Albertyn J, Pohl CH. 2018. Iron at the centre of Candida albicans interactions. Front Cell Infect Microbiol 8:185. doi:10.3389/fcimb.2018.00185 29922600 PMC5996042

[6] Hood MI, Skaar EP. 2012. Nutritional immunity: transition metals at the pathogen-host interface. Nat Rev Microbiol 10:525–537. doi:10.1038/nrmicro2836 22796883 PMC3875331

[7] Murdoch CC, Skaar EP. 2022. Nutritional immunity: the battle for nutrient metals at the host-pathogen interface. Nat Rev Microbiol 20:657–670. doi:10.1038/s41579-022-00745-6 35641670 PMC9153222

[8] Almeida RS, Brunke S, Albrecht A, Thewes S, Laue M, Edwards JE, Filler SG, Hube B. 2008. The hyphal-associated adhesin and invasin Als3 of Candida albicans mediates iron acquisition from host ferritin. PLoS Pathog 4:e1000217. doi:10.1371/journal.ppat.1000217 19023418 PMC2581891

[9] Knight SAB, Vilaire G, Lesuisse E, Dancis A. 2005. Iron acquisition from transferrin by Candida albicans depends on the reductive pathway. Infect Immun 73:5482–5492. doi:10.1128/IAI.73.9.5482-5492.2005 16113264 PMC1231083

[10] Kornitzer D, Roy U. 2020. Pathways of heme utilization in fungi. Biochim Biophys Acta Mol Cell Res 1867:118817. doi:10.1016/j.bbamcr.2020.118817 32777371

[11] Kuznets G, Vigonsky E, Weissman Z, Lalli D, Gildor T, Kauffman SJ, Turano P, Becker J, Lewinson O, Kornitzer D. 2014. A relay network of extracellular heme-binding proteins drives C. albicans iron acquisition from hemoglobin. PLoS Pathog 10:e1004407. doi:10.1371/journal.ppat.1004407 25275454 PMC4183699

[12] Roy U, Yaish S, Weissman Z, Pinsky M, Dey S, Horev G, Kornitzer D. 2022. Ferric reductase-related proteins mediate fungal heme acquisition. Elife 11:e80604. doi:10.7554/eLife.80604 36200752 PMC9635878

[13] Heymann P, Gerads M, Schaller M, Dromer F, Winkelmann G, Ernst JF. 2002. The siderophore iron transporter of Candida albicans (Sit1p/Arn1p) mediates uptake of ferrichrome-type siderophores and is required for epithelial invasion. Infect Immun 70:5246–5255. doi:10.1128/IAI.70.9.5246-5255.2002 12183576 PMC128288

[14] Xu N, Qian K, Dong Y, Chen Y, Yu Q, Zhang B, Xing L, Li M. 2014. Novel role of the Candida albicans ferric reductase gene CFL1 in iron acquisition, oxidative stress tolerance, morphogenesis and virulence. Res Microbiol 165:252–261. doi:10.1016/j.resmic.2014.03.001 24631590

[15] Ramanan N, Wang Y. 2000. A high-affinity iron permease essential for Candida albicans virulence. Science 288:1062–1064. doi:10.1126/science.288.5468.1062 10807578

[16] Navarathna DHMLP, Roberts DD. 2010. Candida albicans heme oxygenase and its product CO contribute to pathogenesis of candidemia and alter systemic chemokine and cytokine expression. Free Radic Biol Med 49:1561–1573. doi:10.1016/j.freeradbiomed.2010.08.020 20800092 PMC2952735

[17] Hameed S, Prasad T, Banerjee D, Chandra A, Mukhopadhyay CK, Goswami SK, Lattif AA, Chandra J, Mukherjee PK, Ghannoum MA, Prasad R. 2008. Iron deprivation induces EFG1 -mediated hyphal development in Candida albicans without affecting biofilm formation . FEMS Yeast Res 8:744–755. doi:10.1111/j.1567-1364.2008.00394.x 18547332

[18] Mochochoko BM, Ezeokoli OT, Sebolai O, Albertyn J, Pohl CH. 2021. Role of the high-affinity reductive iron acquisition pathway of Candida albicans in prostaglandin E2 production, virulence, and interaction with Pseudomonas aeruginosa. Med Mycol 59:869–881. doi:10.1093/mmy/myab015 33862618

[19] Van Genechten W, Vergauwen R, Van Dijck P. 2024. The intricate link between iron, mitochondria and azoles in Candida species. FEBS J 291:3568–3580. doi:10.1111/febs.16977 37846606

[20] Prasad T, Chandra A, Mukhopadhyay CK, Prasad R. 2006. Unexpected link between iron and drug resistance of Candida spp.: iron depletion enhances membrane fluidity and drug diffusion, leading to drug-susceptible cells. Antimicrob Agents Chemother 50:3597–3606. doi:10.1128/AAC.00653-06 16954314 PMC1635214

[21] Puri S, Kumar R, Rojas IG, Salvatori O, Edgerton M. 2019. Iron chelator deferasirox reduces Candida albicans invasion of oral epithelial cells and infection levels in murine oropharyngeal candidiasis. Antimicrob Agents Chemother 63:02152-18. doi:10.1128/AAC.02152-18 PMC6437492 30718249

[22] Kabir S, Sfeir A, de Lange T. 2010. Taking apart Rap1: an adaptor protein with telomeric and non-telomeric functions. Cell Cycle 9:4061–4067. doi:10.4161/cc.9.20.13579 20948311 PMC2995270

[23] Conrad MN, Wright JH, Wolf AJ, Zakian VA. 1990. RAP1 protein interacts with yeast telomeres in vivo: overproduction alters telomere structure and decreases chromosome stability. Cell 63:739–750. doi:10.1016/0092-8674(90)90140-A 2225074

[24] Azad GK, Tomar RS. 2016. The multifunctional transcription factor Rap1: a regulator of yeast physiology. Front Biosci (Landmark Ed) 21:918–930. doi:10.2741/4429 27100480

[25] Dancis A, Roman DG, Anderson GJ, Hinnebusch AG, Klausner RD. 1992. Ferric reductase of Saccharomyses serevisiae: molecular characterization, role in iron uptake, and transcriptional control by iron. Proc Natl Acad Sci U S A 89:3869–3873. doi:10.1073/pnas.89.9.3869 1570306 PMC525592

[26] Kalra S, Peyser R, Ho J, Babbin C, Bohan N, Cortes A, Erley J, Fatima M, Flinn J, Horwitz E, Hsu R, Lee W, Lu V, Narch A, Navas D, Kalu I, Ouanemalay E, Ross S, Sowole F, Specht E, Woo J, Yu K, Coolon JD. 2023. Genome-wide gene expression responses to experimental manipulation of Saccharomyces cerevisiae repressor activator protein 1 (Rap1) expression level. Genomics 115:110625. doi:10.1016/j.ygeno.2023.110625 37068644 PMC10351348

[27] Uemura H, Watanabe-Yoshida M, Ishii N, Shinzato T, Haw R, Aoki Y. 2004. Isolation and characterization of Candida albicans homologue of RAP1, a repressor and activator protein gene in Saccharomyces cerevisiae. Yeast 21:1–10. doi:10.1002/yea.1048 14745778

[28] Yu EY, Yen WF, Steinberg-Neifach O, Lue NF. 2010. Rap1 in Candida albicans: an unusual structural organization and a critical function in suppressing telomere recombination. Mol Cell Biol 30:1254–1268. doi:10.1128/MCB.00986-09 20008550 PMC2820896

[29] Wang WH, Lai TX, Wu YC, Chen ZT, Tseng KY, Lan CY. 2022. Associations of Rap1 with cell wall integrity, biofilm formation, and virulence in Candida albicans. Microbiol Spectr 10:e03285-22. doi:10.1128/spectrum.03285-22 36416583 PMC9769648

[30] Ding C, Festa RA, Sun TS, Wang ZY. 2014. Iron and copper as virulence modulators in human fungal pathogens. Mol Microbiol 93:10–23. doi:10.1111/mmi.12653 24851950

[31] Gerwien F, Skrahina V, Kasper L, Hube B, Brunke S. 2018. Metals in fungal virulence. FEMS Microbiol Rev 42:fux050. doi:10.1093/femsre/fux050 29069482 PMC5812535

[32] Hsu PC, Yang CY, Lan CY. 2011. Candida albicans Hap43 is a repressor induced under low-iron conditions and is essential for iron-responsive transcriptional regulation and virulence. Eukaryot Cell 10:207–225. doi:10.1128/EC.00158-10 21131439 PMC3067405

[33] Mogavero S, Höfs S, Lauer AN, Müller R, Brunke S, Allert S, Gerwien F, Groth S, Dolk E, Wilson D, Gutsmann T, Hube B. 2022. Candidalysin is the hemolytic factor of Candida albicans Toxins (Basel) 14:874. doi:10.3390/toxins14120874 36548771 PMC9785678

[34] Luo G, Samaranayake LP, Yau JY. 2001. Candida species exhibit differential in vitro hemolytic activities. J Clin Microbiol 39:2971–2974. doi:10.1128/JCM.39.8.2971-2974.2001 11474025 PMC88272

[35] Amorim-Vaz S, Tran VDT, Pradervand S, Pagni M, Coste AT, Sanglard D. 2015. RNA enrichment method for quantitative transcriptional analysis of pathogens in vivo applied to the fungus Candida albicans. mBio 6:e00942-15. doi:10.1128/mBio.00942-15 26396240 PMC4600103

[36] Mochon AB, Ye J, Kayala MA, Wingard JR, Clancy CJ, Nguyen MH, Felgner P, Baldi P, Liu H. 2010. Serological profiling of a Candida albicans protein microarray reveals permanent host-pathogen interplay and stage-specific responses during candidemia. PLoS Pathog 6:e1000827. doi:10.1371/journal.ppat.1000827 20361054 PMC2845659

[37] Roy U, Kornitzer D. 2019. Heme-iron acquisition in fungi. Curr Opin Microbiol 52:77–83. doi:10.1016/j.mib.2019.05.006 31265986

[38] Pendrak ML, Chao MP, Yan SS, Roberts DD. 2004. Heme oxygenase in Candida albicans is regulated by hemoglobin and is necessary for metabolism of exogenous heme and hemoglobin to alpha-biliverdin. J Biol Chem 279:3426–3433. doi:10.1074/jbc.M311550200 14615478

[39] Lee Y, Puumala E, Robbins N, Cowen LE. 2021. Antifungal drug resistance: molecular mechanisms in Candida albicans and beyond. Chem Rev 121:3390–3411. doi:10.1021/acs.chemrev.0c00199 32441527 PMC8519031

[40] Morais Vasconcelos Oliveira J, Conceição Oliver J, Latércia Tranches Dias A, Barbosa Padovan AC, Siqueira Caixeta E, Caixeta Franco Ariosa M. 2021. Detection of ERG11 overexpression in Candida albicans isolates from environmental sources and clinical isolates treated with inhibitory and subinhibitory concentrations of fluconazole. Mycoses 64:220–227. doi:10.1111/myc.13208 33176021

[41] Wu Y, Gao N, Li C, Gao J, Ying C. 2017. A newly identified amino acid substitution T123I in the 14α-demethylase (Erg11p) of Candida albicans confers azole resistance. FEMS Yeast Res 17:fox012. doi:10.1093/femsyr/fox012 28334124

[42] Martel CM, Parker JE, Bader O, Weig M, Gross U, Warrilow AGS, Rolley N, Kelly DE, Kelly SL. 2010. Identification and characterization of four azole-resistant erg3 mutants of Candida albicans. Antimicrob Agents Chemother 54:4527–4533. doi:10.1128/AAC.00348-10 20733039 PMC2976150

[43] Morio F, Pagniez F, Lacroix C, Miegeville M, Le Pape P. 2012. Amino acid substitutions in the Candida albicans sterol Δ5,6-desaturase (Erg3p) confer azole resistance: characterization of two novel mutants with impaired virulence. J Antimicrob Chemother 67:2131–2138. doi:10.1093/jac/dks186 22678731

[44] Ivnitski-Steele I, Holmes AR, Lamping E, Monk BC, Cannon RD, Sklar LA. 2009. Identification of Nile red as a fluorescent substrate of the Candida albicans ATP-binding cassette transporters Cdr1p and Cdr2p and the major facilitator superfamily transporter Mdr1p. Anal Biochem 394:87–91. doi:10.1016/j.ab.2009.07.001 19577533 PMC2739806

[45] Bullen JJ, Rogers HJ, Spalding PB, Ward CG. 2006. Natural resistance, iron and infection: a challenge for clinical medicine. J Med Microbiol 55:251–258. doi:10.1099/jmm.0.46386-0 16476787

[46] Winterbourn CC. 1995. Toxicity of iron and hydrogen peroxide: the Fenton reaction. Toxicol Lett 82–83:969–974. doi:10.1016/0378-4274(95)03532-x 8597169

[47] Biswas K, Rieger KJ, Morschhäuser J. 2003. Functional analysis of CaRAP1, encoding the Repressor/activator protein 1 of Candida albicans. Gene 307:151–158. doi:10.1016/s0378-1119(03)00456-6 12706897

[48] Wang WH, Chen HY, Chen SY, Lan CY. 2024. Transcriptional profiling reveals the role of Candida albicans Rap1 in oxidative stress response. Biosci Rep 44:BSR20240689. doi:10.1042/BSR20240689 39575984 PMC11667096

[49] Demain AL. 1972. Riboflavin oversynthesis. Annu Rev Microbiol 26:369–388. doi:10.1146/annurev.mi.26.100172.002101 4567521

[50] Massey V. 2000. The chemical and biological versatility of riboflavin. Biochem Soc Trans 28:283–296. 10961912

[51] Dietl AM, Meir Z, Shadkchan Y, Osherov N, Haas H. 2018. Riboflavin and pantothenic acid biosynthesis are crucial for iron homeostasis and virulence in the pathogenic mold Aspergillus fumigatus. Virulence 9:1036–1049. doi:10.1080/21505594.2018.1482181 30052132 PMC6068542

[52] Gnandt E, Dörner K, Strampraad MFJ, de Vries S, Friedrich T. 2016. The multitude of iron-sulfur clusters in respiratory complex I. Biochim Biophys Acta 1857:1068–1072. doi:10.1016/j.bbabio.2016.02.018 26944855

[53] Demuyser L, Palmans I, Vandecruys P, Van Dijck P. 2020. Molecular elucidation of riboflavin production and regulation in Candida albicans, toward a novel antifungal drug target. mSphere 5:e00714-20. doi:10.1128/mSphere.00714-20 32759338 PMC7407072

[54] Worst DJ, M. Gerrits M, Vandenbroucke-Grauls CMJE, Kusters JG. 1998. Helicobacter pylori ribBA -mediated riboflavin production is involved in iron acquisition . J Bacteriol 180:1473–1479. doi:10.1128/JB.180.6.1473-1479.1998 9515916 PMC107047

[55] Crossley RA, Gaskin DJH, Holmes K, Mulholland F, Wells JM, Kelly DJ, van Vliet AHM, Walton NJ. 2007. Riboflavin biosynthesis is associated with assimilatory ferric reduction and iron acquisition by Campylobacter jejuni. Appl Environ Microbiol 73:7819–7825. doi:10.1128/AEM.01919-07 17965203 PMC2168145

[56] Cain TJ, Smith AT. 2021. Ferric iron reductases and their contribution to unicellular ferrous iron uptake. J Inorg Biochem 218:111407. doi:10.1016/j.jinorgbio.2021.111407 33684686 PMC8035299

[57] Coves J, Fontecave M. 1993. Reduction and mobilization of iron by a NAD(P)H:flavin oxidoreductase from Escherichia coli. Eur J Biochem 211:635–641. doi:10.1111/j.1432-1033.1993.tb17591.x 8436123

[58] Knight SAB, Lesuisse E, Stearman R, Klausner RD, Dancis A. 2022. Reductive iron uptake by Candida albicans: role of copper, iron and the TUP1 regulator. Microbiol 148:29–40. doi:10.1099/00221287-148-1-29 11782496

[59] Ramírez-Zavala B, Krüger I, Dunker C, Jacobsen ID, Morschhäuser J. 2022. The protein kinase Ire1 has a Hac1-independent essential role in iron uptake and virulence of Candida albicans. PLoS Pathog 18:e1010283. doi:10.1371/journal.ppat.1010283 35108336 PMC8846550

[60] van Wijlick L, Znaidi S, Hernández-Cervantes A, Basso V, Bachellier-Bassi S, d’Enfert C. 2022. Functional portrait of Irf1 (Orf19.217), a regulator of morphogenesis and iron homeostasis in Candida albicans. Front Cell Infect Microbiol 12:960884. doi:10.3389/fcimb.2022.960884 36004328 PMC9393397

[61] Braun BR, Johnson AD. 1997. Control of filament formation in Candida albicans by the transcriptional repressor TUP1. Science 277:105–109. doi:10.1126/science.277.5322.105 9204892

[62] Nobile CJ, Fox EP, Nett JE, Sorrells TR, Mitrovich QM, Hernday AD, Tuch BB, Andes DR, Johnson AD. 2012. A recently evolved transcriptional network controls biofilm development in Candida albicans. Cell 148:126–138. doi:10.1016/j.cell.2011.10.048 22265407 PMC3266547

[63] Park YN, Morschhäuser J. 2005. Candida albicans MTLalpha tup1Delta mutants can reversibly switch to mating-competent, filamentous growth forms. Mol Microbiol 58:1288–1302. doi:10.1111/j.1365-2958.2005.04898.x 16313617

[64] Zhao R, Lockhart SR, Daniels K, Soll DR. 2002. Roles of TUP1 in switching, phase maintenance, and phase-specific gene expression in Candida albicans. Eukaryot Cell 1:353–365. doi:10.1128/EC.1.3.353-365.2002 12455984 PMC118011

[65] Blankenship JR, Mitchell AP. 2011. Candida albicans adds more weight to iron regulation. Cell Host Microbe 10:93–94. doi:10.1016/j.chom.2011.08.001 21843865

[66] Baek YU, Li M, Davis DA. 2008. Candida albicans ferric reductases are differentially regulated in response to distinct forms of iron limitation by the Rim101 and CBF transcription factors. Eukaryot Cell 7:1168–1179. doi:10.1128/EC.00108-08 18503007 PMC2446673

[67] Chen C, Pande K, French SD, Tuch BB, Noble SM. 2011. An iron homeostasis regulatory circuit with reciprocal roles in Candida albicans commensalism and pathogenesis. Cell Host Microbe 10:118–135. doi:10.1016/j.chom.2011.07.005 21843869 PMC3165008

[68] Lan CY, Rodarte G, Murillo LA, Jones T, Davis RW, Dungan J, Newport G, Agabian N. 2004. Regulatory networks affected by iron availability in Candida albicans. Mol Microbiol 53:1451–1469. doi:10.1111/j.1365-2958.2004.04214.x 15387822

[69] Singh RP, Prasad HK, Sinha I, Agarwal N, Natarajan K. 2011. Cap2-HAP complex is a critical transcriptional regulator that has dual but contrasting roles in regulation of iron homeostasis in Candida albicans. J Biol Chem 286:25154–25170. doi:10.1074/jbc.M111.233569 21592964 PMC3137088

[70] Chen C, Noble SM. 2012. Post-transcriptional regulation of the Sef1 transcription factor controls the virulence of Candida albicans in its mammalian host. PLoS Pathog 8:e1002956. doi:10.1371/journal.ppat.1002956 23133381 PMC3486892

[71] Manns JM, Mosser DM, Buckley HR. 1994. Production of a hemolytic factor by Candida albicans. Infect Immun 62:5154–5156. doi:10.1128/iai.62.11.5154-5156.1994 7927798 PMC303238

[72] Watanabe T, Takano M, Murakami M, Tanaka H, Matsuhisa A, Nakao N, Mikami T, Suzuki M, Matsumoto T. 1999. Characterization of a haemolytic factor from Candida albicans. Microbiology (Reading, Engl) 145:689–694. doi:10.1099/13500872-145-3-689 10217503

[73] Moyes DL, Wilson D, Richardson JP, Mogavero S, Tang SX, Wernecke J, Höfs S, Gratacap RL, Robbins J, Runglall M, et al.. 2016. Candidalysin is a fungal peptide toxin critical for mucosal infection. Nature 532:64–68. doi:10.1038/nature17625 27027296 PMC4851236

[74] Richardson JP, Mogavero S, Moyes DL, Blagojevic M, Krüger T, Verma AH, Coleman BM, De La Cruz Diaz J, Schulz D, Ponde NO, Carrano G, Kniemeyer O, Wilson D, Bader O, Enoiu SI, Ho J, Kichik N, Gaffen SL, Hube B, Naglik JR. 2018. Processing of Candida albicans Ece1p is critical for candidalysin maturation and fungal virulence. mBio 9:e02178-17. doi:10.1128/mBio.02178-17 29362237 PMC5784256

[75] Russell CM, Schaefer KG, Dixson A, Gray ALH, Pyron RJ, Alves DS, Moore N, Conley EA, Schuck RJ, White TA, Do TD, King GM, Barrera FN. 2022. The Candida albicans virulence factor candidalysin polymerizes in solution to form membrane pores and damage epithelial cells. Elife 11:e75490. doi:10.7554/eLife.75490 36173096 PMC9522247

[76] Pappas PG, Kauffman CA, Andes DR, Clancy CJ, Marr KA, Ostrosky-Zeichner L, Reboli AC, Schuster MG, Vazquez JA, Walsh TJ, Zaoutis TE, Sobel JD. 2016. Clinical practice guideline for the management of candidiasis: 2016 update by the Infectious Diseases Society of America. Clin Infect Dis 62:e1–50. doi:10.1093/cid/civ933 26679628 PMC4725385

[77] Whaley SG, Berkow EL, Rybak JM, Nishimoto AT, Barker KS, Rogers PD. 2020. Azole antifungal resistance in Candida albicans and emerging non-albicans Candida species. Front Microbiol 7:231851. doi:10.3389/fmicb.2016.02173 PMC5226953 28127295

[78] Jordá T, Puig S. 2020. Regulation of ergosterol biosynthesis in Saccharomyces cerevisiae Genes (Basel) 11:795. doi:10.3390/genes11070795 32679672 PMC7397035

[79] Liu TT, Lee REB, Barker KS, Lee RE, Wei L, Homayouni R, Rogers PD. 2005. Genome-wide expression profiling of the response to azole, polyene, echinocandin, and pyrimidine antifungal agents in Candida albicans. Antimicrob Agents Chemother 49:2226–2236. doi:10.1128/AAC.49.6.2226-2236.2005 15917516 PMC1140538

[80] Kohli A, Smriti NFN, Mukhopadhyay K, Rattan A, Prasad R. 2002. In vitro low-level resistance to azoles in Candida albicans is associated with changes in membrane lipid fluidity and asymmetry . Antimicrob Agents Chemother 46:1046–1052. doi:10.1128/AAC.46.4.1046-1052.2002 11897588 PMC127087

[81] Mukhopadhyay K, Kohli A, Prasad R. 2002. Drug susceptibilities of yeast cells are affected by membrane lipid composition. Antimicrob Agents Chemother 46:3695–3705. doi:10.1128/AAC.46.12.3695-3705.2002 12435664 PMC132749

[82] Shanmugam R, Majee P, Shi W, Ozturk MB, Vaiyapuri TS, Idzham K, Raju A, Shin SH, Fidan K, Low J-L, et al.. 2024. Iron-(Fe3+)-dependent reactivation of telomerase drives colorectal cancers. Cancer Discov 14:1940–1963. doi:10.1158/2159-8290.CD-23-1379 38885349 PMC11450372

[83] Zhang C. 2014. The correlation between iron homeostasis and telomere maintenance. Front Biol 9:347–355. doi:10.1007/s11515-014-1327-x

[84] Diotti R, Esposito M, Shen CH. 2021. Telomeric and sub-telomeric structure and implications in fungal opportunistic pathogens. Microorganisms 9:1405. doi:10.3390/microorganisms9071405 34209786 PMC8305976

[85] Livak KJ, Schmittgen TD. 2001. Analysis of relative gene expression data using real-time quantitative PCR and the 2(-delta delta C(T)) method. Methods 25:402–408. doi:10.1006/meth.2001.1262 11846609

[86] Schatzman SS, Peterson RL, Teka M, He B, Cabelli DE, Cormack BP, Culotta VC. 2020. Copper-only superoxide dismutase enzymes and iron starvation stress in Candida fungal pathogens. J Biol Chem 295:570–583. doi:10.1074/jbc.RA119.011084 31806705 PMC6956530

[87] McDonagh AF, Palma LA. 1980. Preparation and properties of crystalline biliverdin IX alpha. Simple methods for preparing isomerically homogeneous biliverdin and [14C[biliverdin by using 2,3-dichloro-5,6-dicyanobenzoquinone. Biochem J 189:193–208. doi:10.1042/bj1890193 7458909 PMC1161990

[88] Clinical and Laboratory Standards Institute (CLSI). 2008. Reference method for broth dilution antifungal susceptibility testing of yeast. In CLSI document M27-A3. CLSI, Wayne, PA.

