# Supplementary material for: Deletion of RAP1 affects iron homeostasis, azole resistance, and virulence in Candida albicans
Source: mSphere. 2025 Apr 23;10(5):e00155-25. doi: 10.1128/msphere.00155-25 (PMC12108065; doi:10.1128/msphere.00155-25)
Supplement: Supplemental material — Fig. S1, Table S1 and S2 captions, Tables S3 and S4, and supplemental text. [file msphere.00155-25-s0001.docx]

**Supplemental Figure**

**Fig S1** Differential expression of the *FTR1* and *FTR2* genes under different iron conditions. Iron pre-starved cells were subcultured in YPD (“high iron”) and YPD supplemented with 200 μM BPS (“low iron’) media and grown at 30℃ for 5 hours. The *FTR1 and FTR2* transcripts were determined by real-time qPCR. The *ACT1* transcripts were used as endogenous control. Data are collected from three independent experiments and presented as the mean ± standard deviation (SD). *, *P* < 0.05.; ***, *P* < 0.001.

**Supplemental Tables**

**Table S1**. List of differentially expressed genes (DEGs).

Table S1 includes DEGs from RNA-seq analysis, comparing the *rap1*Δ/Δ and wild-type strains in YPD supplemented with 200 μM BPS (“low iron”) and YPD (“high iron”) and medium, respectively. Table S1 is provided in a separate Excel file.

**Table S2.** GO categories.

Table S2 includes GO analysis data from all the DEGs listed in Table S1. Table S2 is provided in a separate Excel file.

**Table S3. *C*. *albicans* strains used in this study**

| Strain name | Genotype | Source |
| --- | --- | --- |
| SC5314 | Wild type | 1 |
| *rap1*Δ/Δ | *rap1*Δ::*FRT*/*rap1*Δ::*FRT* | 2 |
| *RAP1*R | *rap1*Δ::*RAP1*-*FRT*/*rap1*Δ::*RAP1*-*FRT* | 2 |

1 Gillum AM, Tsay EY, Kirsch DR. 1984. Isolation of the *Candida albicans* gene for orotidine-5'-phosphate decarboxylase by complementation of *S*. *cerevisiae* ura3 and *E*. *coli* pyrF mutations. Mol Gen Genet 198:179-182.

2 Wang WH, Lai TX, Wu YC, Chen ZT, Tseng KY, Lan CY. 2022. Associations of Rap1 with cell wall integrity, biofilm formation, and virulence in *Candida albicans*. Microbiol Spectr 10:e03285-22.

**Table S4. Primers used in this study**

| Primers | Sequences (5' to 3') |
| --- | --- |
| ACT1-F | ATACTCTGTCTGGATTGGTGGTTCT |
| ACT1-R | TTTTGAAATCCACATTTGTTGGA |
| FTR1-F | GCGTGAAGGTTTAGAAGCTGTTG |
| FTR1-R | GGGAAAGAGGTGGCTGGACTA |
| CFL2-F | GCTAGCAAAACCCTGTCTACGAAA |
| CFL2-R | AGCTTTACCTGTGCTGGAGGAT |
| FET31-F | TGCCGGTGTCTTAGGTTTAGCC |
| FET31-R | TGACGATTTCGTTATCTTCTTC |
| HMX1-F | GGCCTTGTACAGACAGCTTGAA |
| HMX1-R | TCTCCGGTTTCCAAACTTGCT |
| PGA7-F | TTCCTCGATGCTTTCCACTGCC |
| PGA7-R | ATGAGCCTGTAGATGACGAGCC |
| RBT5-F | TGCTCGCCTTATCCTTATTGTC |
| RBT5-R | GTTGATGGAAGCGGTTTTAGC |
| SIT1-F | GGTGGTATGTGTTTGATGGGATT |
| SIT1-R | TTTGGGCACTGACATTAATTGG |
| ERG11-F | ACCATTTGGTGGTGGTAGACA |
| ERG11-R | AGGGTCAGGCACTTTATAACCA |
| ERG3-F | TACCGCTTGTCACACTGTCC |
| ERG3-R | CCCAAAGAGTAGTGAATTGACCG |
| MDR1-F | TTACCTGAAACTTTTGGCAAAACA |
| MDR1-R | ACTTGTGATTCTGTCGTTACCG |
| CDR1-F | ATTCTAAGATGTCGTCGCAAGATG |
| CDR1-R | AGTTCTGGCTAAATTCGTAATGTTTTC |

**Supplemental Methods**

**A. RNA-seq and data analysis.** Total RNA was extracted from three biological replicates of each strain using NautiaZ Bacteria/Fungi RNA Mini Kit (Nautia Gene, Taipei, Taiwan). The purified RNA was used to prepare the sequencing library with the TruSeq Stranded mRNA Library Prep Kit (Illumina, San Diego, CA, USA). Briefly, mRNA was purified by oligo(dT)-coupled magnetic beads, fragmented into small pieces at elevated temperatures, and first-strand cDNA was synthesized. After generating double-strand cDNA and adenylation on 3’-ends of DNA fragments, adaptors were ligated to the fragments. The resulting products were PCR-enriched and purified using the AMPure XP system (Beckman Coulter, Beverly, USA). The libraries were analyzed, qualified and then sequenced on an Illumina NovaSeq platform with 150 bp paired-end reads by Genomics BioSci & Tech Co. (New Taipei City, Taiwan).

The processed sequence reads were aligned to the reference *C. albicans* genome SC5314_A22 using HISAT2 (1). The raw read counts from RNA-seq were used to perform transcriptome analysis. The differential expression genes (DEGs) analysis was performed with the PyDESeq2 package (version 0.4.12) in the Python environment using the default parameter based on the workflow description of the PyDESeq2 (https://pydeseq2.readthedocs.io/en/stable/auto_examples/plot_step_by_step.html#sphx-glr-auto-examples-plot-step-by-step-py) with some modifications. To adjust the differences in sequencing depth across samples, the read counts were normalized to the size factors derived from median ratio of gene counts relative to a pseudo-reference (the geometric mean of gene counts across all samples). To model the variability of the read counts across the biological replicates, the normalized read counts were further fit to the trend curve derived from the dispersion estimation based on negative binomial distribution. Log2 Fold Change (LFC) estimation was conducted by calculation of log2 fold change between conditions and LFC shrinkage was applied to reduce the noise. The Wald test was used to compute the *p*-values (*P*) for each gene. To control the false discovery rate (FDR) for multiple tests, the adjusted *p*-values (adjusted *P*) were computed by the Benjamini-Hochberg method. DEGs were defined by cut-off at the adjusted *P* of 0.05 and at least 2-fold change. Volcano plots were generated in SRplot with *p*-value <0.05 and fold change threshold of 2. GO enrichment analysis was carried out using the GO TermFinder tool from the Candida Genome Database (2). The significant thresholds for GO enrichment analysis were set at *P* < 0.1.

**B. Virulence assay****.** Female BALB/c mice were obtained from BioLASCO Taiwan Co., Ltd. (Taipei, Taiwan) and housed (5 mice per cage) for 2 weeks before experiments (3). A *C. albicans* cell suspension (10^6^ cells) was injected via the lateral tail vein. The infected mice were inspected for any signs of distress and survival twice per day for 2 weeks. The animal studies were approved by the Institutional Animal Care and Use Committees of National Chung Hsing University, Taiwan (IACUC No. 113-053). The log-rank test was used to assess the differences in survival between groups of mice.

On the second day post-infection, a group of mice were euthanized, and the kidneys were extracted for fungal burden analysis and histological examination (3). Samples were observed using a ZEISS Apotome 3 microscope with 10x, 20x, and 63x objectives. Immersion oil was applied to the 63x objective to enhance resolution. Images were processed using ZEISS ZEN software, with scale bars of 100 μm, 50 μm, and 20 μm corresponding to 10x, 20x, and 63x magnifications, respectively.

References for Supplemental Methods

1. Kim D, Paggi JM, Park C, Bennett C, Salzberg SL. 2019. Graph-based genome alignment and genotyping with HISAT2 and HISAT-genotype. Nat Biotechnol 37: 907–15.
2. Boyle EI, Weng S, Gollub J, Jin H, Botstein D, Cherry JM, Sherlock G. 2004. GO:: TermFinder—open source software for accessing Gene Ontology information and finding significantly enriched Gene Ontology terms associated with a list of genes. Bioinformatics 20:3710-3715.

3. Hsu PC, Yang CY, Lan CY. 2011. *Candida albicans* Hap43 is a repressor induced under low-iron conditions and is essential for iron-responsive transcriptional regulation and virulence. Eukaryot Cell 10:207-225.
